# Thioredoxin, oxidative stress, cancer and aging

**DOI:** 10.1186/2046-2395-1-4

**Published:** 2012-09-03

**Authors:** Lisa C Flores, Melanie Ortiz, Sara Dube, Gene B Hubbard, Shuko Lee, Adam Salmon, Yiqiang Zhang, Yuji Ikeno

**Affiliations:** 1 Barshop Institute for Longevity and Aging Studies, The University of Texas Health Science Center at San Antonio, San Antonio, TX, 78229, USA; 2 Department of Pathology, The University of Texas Health Science Center at San Antonio, San Antonio, TX, 78229, USA; 3 Molecular Medicine, The University of Texas Health Science Center at San Antonio, San Antonio, TX, 78229, USA; 4 Research Service, Audie L. Murphy VA Hospital South Texas Veterans Health Care System, San Antonio, TX, 78229, USA; 5 Geriatric Research and Education Center, Audie L. Murphy VA Hospital South Texas Veterans Health Care System, San Antonio, TX, 78229, USA

**Keywords:** Thioredoxin, Transgenic mouse, Knockout mouse, Oxidative stress, Cancer, aging

## Abstract

The Free Radical or Oxidative Stress Theory of Aging is one of the most popular theories in aging research and has been extensively studied over the past several decades. However, recent evidence using transgenic/knockout mice that overexpress or down-regulate antioxidant enzymes challenge the veracity of this theory since the animals show no increase or decrease in lifespan. These results seriously call into question the role of oxidative damage/stress in the aging process in mammals. Therefore, the theory requires significant modifications if we are to understand the relationship between aging and the regulation of oxidative stress. Our laboratory has been examining the impacts of thioredoxins (Trxs), in the cytosol and mitochondria, on aging and age-related diseases. Our data from mice that are either up-regulating or down-regulating Trx in different cellular compartments, that is, the cytosol or mitochondria, could shed some light on the role of oxidative stress and its pathophysiological effects. The results generated from our lab and others may indicate that: 1) changes in oxidative stress and the redox state in the cytosol, mitochondria or nucleus might play different roles in the aging process; 2) the role of oxidative stress and redox state could have different pathophysiological consequences in different tissues/cells, for example, mitotic vs. post-mitotic; 3) oxidative stress could have different pathophysiological impacts in young and old animals; and 4) the pathophysiological roles of oxidative stress and redox state could be controlled through changes in redox-sensitive signaling, which could have more diverse effects on pathophysiology than the accumulation of oxidative damage to various molecules. To critically test the role of oxidative stress on aging and age-related diseases, further study is required using animal models that regulate oxidative stress levels differently in each cellular compartment, each tissue/organ, and/or at different stages of life (young, middle and old) to change redox sensitive signaling pathways.

## Review

### Oxidative stress theory of aging

The Free Radical or Oxidative Stress Theory of Aging is one of the most popular theories in aging research and has been extensively studied over the past several decades. This theory is based on the fact that cells exist in a chronic state of oxidative stress resulting from an imbalance between pro-oxidants and antioxidants. Because of this imbalance, which occurs as a consequence of aerobic metabolism, it is proposed that an accumulation of oxidative damage occurs with age in a variety of macromolecules within the cell. This steady state of increased oxidative damage is proposed to be an important factor in the age-related increase in pathology and the progressive decline in the functional efficiency of various cellular processes [1-3].

One consistent line of evidence to support the oxidative stress hypothesis of aging is the large amount of data that has shown an age-related increase of oxidative damage in a variety of molecules (lipids, proteins and DNA) in organisms ranging from invertebrates to humans [1,4-10]. Another strong line of evidence comes from manipulation studies that increase lifespan. Calorie restriction (CR), which extends lifespan and delays aging in various species, has been shown to reduce the level of oxidative damage in tissues as measured by a decrease in lipofuscin [11-13], lipid peroxidation [13-18], protein oxidation [10,19-23] and DNA oxidation [24-26]. Subsequently, CR mice have also been shown to be more resistant to oxidative stress [2]. Mutations in the insulin/IGF-1 signaling pathways (*age-1daf2*, and *daf16* mutants) of *Caenorhabditis elegans* showed an increase in lifespan*,* which was also correlated with increased resistance to oxidative stress [27] and reduced oxidative damage [28]. More recently, several genetic mouse models of longevity have been reported, for example, Ames and Snell dwarf mice, *p66*^*sch−/−*^ mice, and *Igf1r*^*+/−*^ female mice [29], and the increased lifespan in these models has been correlated to increased resistance to oxidative stress [30]. Thus, the observations that experimental manipulations that increase lifespan in rodents and invertebrates are correlated to increased resistance to oxidative stress or reduced oxidative damage have provided strong evidence in support of the oxidative stress theory of aging. However, all the experimental manipulations that increase lifespan also alter processes beyond oxidative stress/damage; therefore, the increased longevity in these animal models could arise through other mechanisms. A recent study using naked mole-rats, which have a lifespan approaching 30 years, showed an increased amount of oxidative damage compared to the short-lived mouse [31] and calls into question the role of oxidative damage in aging.

### Transgenic (TG)/knockout (KO) animal models for the free radical/oxidative stress theory of aging

Transgenic/knockout animals provide investigators with a unique system for studying the underlying mechanisms of various biological processes and have been used to conduct numerous studies to test the various theories of aging. The most direct test of the oxidative stress hypothesis of aging is to alter the accumulation of oxidative damage and determine its effect on aging/lifespan. Over the past two decades, investigators have used *Drosophila* or mice with genetic alterations in the antioxidant defense system as a strategy to alter the age-related accumulation of oxidative damage. Data from these studies have the potential to establish a causative role for oxidative stress/damage in aging. However, studies between *Drosophila* and mice have shown inconsistent results, raising the possibility that differences in species might affect the outcome of a genetic manipulation on lifespan.

#### *Drosophila*

In the initial studies using P-element mediated transformation, researchers introduced either Cu/Zn superoxide dismutase (SOD) [32-34] or catalase [35] into *Drosophila*; the survival of the resulting flies was not significantly different from their wild-type controls. Subsequently, Orr and Sohal [36] reported that overexpression of both Cu/ZnSOD and catalase significantly increased (from 14 to 34%) maximum lifespan and increased the mortality rate doubling time from 20% to 37%. Additionally, these transgenic lines of *Drosophila* had significantly lower levels of protein and DNA oxidation [36,37]. However, these studies are complicated because the placement of P-elements has been shown to alter lifespan independently [38,39]. Using a combined total of over 90,000 flies to minimize the problem of P-element insertion, Orr *et al.*[40] found that the overexpression of both Cu/ZnSOD and catalase had no beneficial effects on survivorship and was in fact associated with slightly decreased lifespans in long-lived *Drosophila* strains. Investigators have also used inducible systems to overexpress antioxidant genes in *Drosophila* to circumvent the problem of P-element insertion. These studies have shown that overexpression of Cu/ZnSOD or MnSOD increased the lifespan of *Drosophila*, but overexpression of catalase had no benefit [41,42]. Parkes *et al.*[43] showed that the induced overexpression of Cu/ZnSOD selectively targeted to motor neurons resulted in a 40% increase in lifespan in *Drosophila* and increased resistance to oxidative stress. Two groups studied the effect of inducing the overexpression of methionine sulfoxide reductase A (MsrA), which repairs oxidized methionine, in *Drosophila*. Chavous *et al.*[44,45] showed that overexpressing MsrA 3- to 7-fold resulted in a 32% to 39% extension in lifespan, while Ruan* et al.*[46] showed that the overexpression of MsrA in the nervous system of *Drosophila* increased the fly’s lifespan and resistance to oxidative stress.

#### Mice

Several groups have genetically altered various components of the antioxidant defense system in mice. In 1987, Epstein’s laboratory produced a transgenic mouse that overexpressed human Cu/ZnSOD 1.6- to 6-fold in various tissues [47]. These transgenic mice were more resistant to cerebral ischemia [48,49], but their lifespan was the same as the wild-type mice [50]. Survival studies with various transgenic mice have also shown negative results. Our group has conducted survival studies using mice that overexpress Cu/ZnSOD, MnSOD, catalase (in peroxisomes), Cu/ZnSOD and MnSOD, and Cu/ZnSOD and catalase; these transgenic mice were not observed to have an increased lifespan compared to their wild-type littermates [51]. On the other hand, transgenic mice that overexpressed catalase in mitochondria showed an increase in lifespan, which was associated with reduced oxidative damage [52]. In contrast, Shriner *et al.*[52] and Richardson’s laboratory showed that the overexpression of catalase in the cytosol (peroxisomes) did not significantly increase lifespan. Therefore, overexpression of antioxidant enzymes does not appear to increase the lifespan of mice, except for the study by Schriner *et al.*[52], which indicates that altering the antioxidant defense system in mitochondria may be more important than in the cytosol.

There is also considerable information on the effect of under-expressing antioxidant enzymes on aging.

Our group has studied the effect of reduced expression of MnSOD on aging and pathology using mice heterozygous for the *Sod2* gene (*Sod2*^*+/−*^ mice). The *Sod2*^*+/−*^ mice showed higher levels of DNA oxidation and a higher incidence of cancer. However, there was no difference in the lifespan between the *Sod2*^*+/−*^ and wild-type mice [53]. Cu/ZnSOD null mice, which lack Cu/ZnSOD, were shown to have a shorter lifespan compared to the wild-type control mice [54]. The short lifespan of these knockout mice (in the C57BL/6 background) appears to be due to a high incidence (approximately 90%) of hepatocellular carcinoma, which was not observed in wild-type mice in the C57BL/6 genetic background. Our recent studies demonstrated that the survival curves of mice that were deficient in Glutathione peroxidase 1 (GPX1) and MnSOD (including genotypes: *Gpx1*^*−/−*^*Sod2*^*+/−*^*Gpx1*^*+/−*^ x *Sod2*^*+/−*^*Gpx1*^*−/−*^ x *Sod2*^*+/−*^) are essentially the same as wild-type mice. These results are also inconsistent with the oxidative stress theory of aging.

Lethality (either by developmental defects or increased uncommon cancers) by the complete absence of some antioxidant enzymes strongly suggests the essential roles antioxidant enzymes play in maintaining cellular and physiological homeostasis in the body. However, based on the data obtained from transgenic and knockout mice, the changing oxidative damage levels by alterations of major antioxidant enzymes does not seem to have a significant impact on lifespan. More importantly, the study by Schriner *et al.*[52] suggests that changes in oxidative stress and the redox state in different cellular compartments, that is, the cytosol or mitochondria, might play different roles in the aging process. Additionally, the mitochondria are a major source of reactive oxygen species (ROS) and substantial evidence suggests that ROS could play important roles in altering cellular signaling pathways [55-57], which could have a more significant impact on aging than the accumulation of oxidative damage.

### Thioredoxin and biological functions

Thioredoxin (Trx) was first recognized in the early 1960s as the reductant for a variety of enzymes. Trx is a small protein (12 kDa) with two redox-active cysteine residues in the active center (Cys-Gly-Pro-Cys) and it has been shown to be reduced by Trx reductase in a NADPH-dependent reaction. In its dithiol form, Trx serves as the reductant for methionine sulfoxide (MetO) and PAPS (3′-phosphoadenosine-5′-phosphosulfate) in yeast and ribonucleotides in *Escherichia coli*[58,59]. Isoforms of Trx have been found in *E. coli*, yeast and mammals. Two Trxs have been identified in humans, one cytosolic (*TRX1*) [60] and one mitochondrial (*TRX2*) [61]. A major role of Trx is as a hydrogen donor for enzymes involved in reductive reactions, for example, ribonucleotide reductase, which reduces ribonucleotides to deoxyribonucleotides for DNA synthesis; peroxiredoxin (Prx), which reduces peroxides [62-64]; and MetO reductase, which reduces methionine sulfoxide in proteins and provides protection against oxidative stress [65-67]. All Trxs catalyze the reduction of disulfides in proteins more efficiently (orders of magnitude faster) than Glutathione (GSH) or dithiothreitol [68]. Therefore, Trx plays an important role in maintaining a reduced environment in the cells through thiol-disulfide exchange reactions and protects cells and tissues from oxidative stress [69]. Trx also plays a major role in thiol-disulfide exchange reactions, which maintains cysteine residues in a reduced state in proteins [68]. Because the thiol-disulfide exchange reaction is rapid and readily reversible, this reaction is ideally suited to control protein function via the redox state. This is particularly important for several transcription factors, such as activator protein 1 (AP-1) and nuclear factor κB (NFκB), which contain cysteine residues [70]. The reduction of cysteine residues increases the DNA binding activity of these transcription factors [71,72], which subsequently induces the expression of target genes. In addition, Trxs have essential roles in the development of mammals because knockout mice null for either Trx1 or Trx2 are embryonically lethal [73,74]. Furthermore, Trx1 has anti-apoptotic effects by binding to apoptosis signal-regulating kinases (ASK) [75].

### Transgenic mice overexpressing Trx1 by β-actin and endogenous promoters

In 1999, Yodoi and colleagues generated transgenic mice overexpressing Trx1 with a transgene containing the human TRX1 cDNA fused to the β–actin promoter (Tg(act-*TRX1*)^+/0^) [69]; they showed that these mice had an extended lifespan compared to their wild-type littermates [76,77]. Our study demonstrated that young and adult mice overexpressing Trx1 had increased resistance to oxidative stress and reduced oxidative damage to proteins and lipids [78]. These results are very exciting for the following reasons: 1) the Tg(act-*TRX1*)^+/0^ mice are the second mouse model (the first one is mCAT mice) to support the oxidative stress theory of aging; and 2) these results could indicate the importance of redox state maintenance by Trx during aging. However, the survival study by Yodoi and colleagues has several possible deficits: 1) the study was conducted under conventional housing conditions, in which the infectious status of these mice could have potentially changed their normal aging process; and 2) the lifespan of the wild-type C57BL/6 mice in the colony was shorter than the C57BL/6 mice raised under barrier conditions, for example, the median lifespan was approximately 23 months of age, which is much shorter than the median lifespan of C57BL/6 mice in the aging colonies in San Antonio (29 to 30 months of age). To examine the effects of increased levels of Trx1 on oxidative stress *in vivo* and its long-term pathophysiological consequences under optimal housing conditions, we have conducted an aging study with Tg(act-*TRX1*)^+/0^ mice [78]. We observed a significant increase in the survival of male Tg(act-*TRX1*)^+/0^ mice compared to the wild-type mice only during the first half of their lifespan. For example, the transgenic mice had a 25% increase in their lifespan in the earlier part of life (75% survival) and a 13% increase in lifespan in the median part of life (50% survival). However, the transgenic mice showed only a 5.5% increase in lifespan in the later part of life (25% survival) and no increase thereafter (10% survival), which was associated with reduced Trx1 overexpression. To further confirm our initial observation, we conducted another survival study using males and females. The male Tg(act-*TRX1*)^+/0^ mice showed a significant increase in lifespan in the very early part of life (90% survival; both cohorts), and only the first male cohort showed a significant increase in lifespan in the early part of life (75% survival) compared to the wild-type mice. However, we found no difference in the lifespan at the median (50%) and the later part of life (25%, and 10% survival) between Tg(act-*TRX1*)^+/0^ and wild-type mice in two male cohorts. Although the Tg(act-*TRX1*)^+/0^ female mice seemed to have an extended lifespan in the earlier part of life (75% survival) compared to wild-type mice, none of the survival parameters were statistically significant [78]. These survival results are partially consistent with the previous study by Yodoi and colleagues that showed that the overexpression of Trx1 significantly increases median and maximum lifespans [76,77]. Differences in results between this and previous studies could be due to different housing conditions of the mouse colony; the mice in our colony showed about a 20% longer (40 months) maximum longevity than mice maintained under conventional housing conditions (approximately 32 months) [77]. Therefore, our survival data did show that overexpression of Trx1 increased only the earlier part of lifespan in males, but no extension was observed in maximum lifespan, which is a question that remains to be answered, that is, why did Trx1 overexpression show an extension of lifespan only in the earlier part of life? These mice were generated with a β-actin promoter to drive the expression of the transgene, which could cause an age-related decrease in the overexpression of the transgene. We observed that the levels of overexpression significantly decreased with age, which was correlated with less reduction in protein oxidation levels [78].

To test if continuous overexpression of Trx1 extends maximum lifespan, we generated an additional line of Trx1 transgenic mice (Trx1Tg) using a fragment of the human genome containing the TRX1 gene (a BAC clone (RP11-427 L11), Children’s Hospital Oakland Research Institute’s (CHORI) BACPAC Resources Center (BPRC), Oakland, CA.) with 8.3 kb and 12.3 kb of the 5′- and 3′-flanking sequences, respectively. We have confirmed that the transgenes in these mice were stably integrated and successfully passed to progeny following the Mendelian ratio for all lines of transgenic mice. We also confirmed that the levels of Trx1 were significantly higher (approximately 20% to 40%) in all the tissues we examined in the Trx1Tg mice, compared to their wild-type littermates, and were similar to the levels of overexpression in the young (4 to 6 months old) Tg(act-*TRX1*)^+/0^ mice. Furthermore, overexpression of Trx1 was maintained (up to 28 to 30 months old), that is, the levels of overexpressed Trx1 did not show any decrease or increase during aging. There were no compensatory changes in the levels of Trx2, glutaredoxin, glutathione or other major antioxidant enzymes. A survival study with Trx1Tg and wild-type mice was conducted and is currently ongoing. The current survival rate of Trx1Tg and wild-type mice is 29.8% and 27.6%, respectively. The earlier parts (75% survival) of lifespan in Trx1Tg and wild-type mice are 707 and 665 days, respectively. The difference (6.3%) is not significant (*P* > 0.05) as determined by the Generalized Wilcoxon test. Mortality rate after 700 days appears to be higher in the Trx1Tg than wild-type mice and no life-extension is currently observed. Therefore, continuous overexpression of Trx1 in mice showed similar effects observed in the Tg(act-*TRX1*)^+/0^ mice, that is, Trx1 overexpression showed some benefits for lifespan only in the earlier part of life.

A possible explanation for these results is that Trx1 could have deleterious effects in older animals because of its anti-apoptotic effect by inhibiting the Apoptosis Signal-Regulating Kinase-1 (ASK1) pathway [75,79]. Our data showed that Tg(act-*TRX1*)^+/0^ mice had higher levels of the ASK1/Trx1 complex and reduced c-Jun N-terminal Kinase (JNK) activation [78]. The major cause of death in these mice was neoplastic disease, especially lymphoma, which is consistent with the end-of-life pathology data from C57BL/6 mice [80]. Interestingly, the end-of-life pathology data showed that Tg(act-*TRX1*)^+/0^ mice had higher incidences of total fatal tumors and fatal lymphoma compared to wild-type mice. This is not a surprising observation because Trx1 has anti-apoptotic action by inhibiting ASK, promoting cell proliferation, and is overexpressed in various cancers, including lymphoma [60,81]. The possible role of Trx1 in tumor development was further confirmed by our study using the brain tumor model, which also showed that increased levels of TRX1 are correlated to increased cell proliferation and reduced cell death in tumors and tumor development (incidence and growth of tumors) [82].

Thus, our studies show that the overexpression of Trx1 in mice had some benefits in the earlier part of lifespan, but did not extend maximum lifespan, possibly due to the higher incidence of fatal tumors compared to wild-type mice. Experimental research on all animals was approved by the Institutional Animal Care and Use Committee (IACUC) of the Department of Veterans Affairs and the University of Texas Health Science Center at San Antonio.

### Oxidative stress theory of aging – future directions of thioredoxins

Although the Free Radical or Oxidative Stress Theory of Aging is generally accepted as an important component of aging and age-related diseases, recent evidence using transgenic/knockout mice overexpressing or down-regulating antioxidant enzymes challenge the veracity of this theory since the animals showed no increase or decrease in lifespan. These results seriously call into question the role of oxidative damage/stress in the aging process in mammals. Thus, significant modifications to the theory are required if we are to understand the relationship between aging and the regulation of oxidative stress. Our laboratory made the interesting observation that the overexpression of Trx1 showed benefits only in the earlier part of life, with an increased incidence of tumors with age. These results led us to the following questions: 1) why did Trx1 overexpression show some beneficial effects on lifespan, while the overexpression of most other antioxidants failed to extend lifespan; and 2) why did Trx1 overexpression show an extension of lifespan only in the earlier part of life?

Several studies demonstrated that the cellular redox state plays an important role in the physiological responses to oxidative stress and aging. We demonstrated that lifespan extension in the earlier part of life in Tg(act-*TRX1*)^+/0^ mice is correlated to increased levels of the Trx1 redox state. The enhanced Trx1 redox state could play an important role in an organism’s ability to better respond to stress by altering the signaling pathways that are dependent on the cellular redox state [83]. The mRNA levels of IL-1β, one of the representative target genes in the NFkB pathway, were significantly lower in the liver tissue from Tg(act-*TRX1*)^+/0^ mice compared to wild-type mice. There is some indication that IL-1β could be involved in the wide-spread systemic inflammatory process. Substantial evidence suggests inflammation plays an important role in aging and, additionally, Tg(act-*TRX1*)^+/0^ mice showed significantly less incidence of inflammatory lesions in the lung (acidophilic macrophage pneumonia) compared to wild-type mice, which occurred mainly in the earlier part of life. Therefore, the reduction in systemic inflammation and suppression of acidophilic macrophage pneumonia, which is a non-neoplastic fatal disease commonly seen in C57BL/6 mice, could be possible contributing factors for the extension of lifespan in the earlier part of life in the Tg(act-*TRX1)*^+/0^ mice. These observations are further confirmed by our most recent study, in which we found that the overexpression of Trx1 can prevent the induction of pro-inflammatory cytokine TNFα in adipose tissue associated with high fat feeding [84]. This reduction of inflammation was associated with preserved glucose tolerance in this model, suggesting that Trx1 prevents insulin resistance through its anti-inflammatory effects. Together, these findings show that Trx1-mediated regulation of the inflammatory state may be a significant regulator of health under various age-related pathophysiological conditions.

The next question is: why does Trx1 overexpression show an extension of lifespan only in the earlier part of life? As described above, we believe that the loss in life extension, or accelerated mortality, in old Trx1 transgenic mice could arise because the overexpression of the TRX1 gene could promote tumor growth, including lymphoma, a major fatal disease in C57BL/6 mice. Trx1 could promote the development of cancer in older animals because of its anti-apoptotic effect by inhibiting the ASK1 pathway and protecting against various stressors [75,78,79]. The Tg(act-*TRX1*)^+/0^ mice had higher levels of the ASK1/Trx1 complex, reduced JNK activation, reduced oxidative damage to lipids and proteins, and had a higher incidence of total fatal tumors and fatal lymphoma compared to wild-type mice. Since Trx1 is overexpressed in various cancers [81] and apoptosis plays important roles in carcinogenesis, overexpression of Trx1 could promote cancer growth by its anti-apoptotic action, protection against oxidative stress, and have deleterious effects in older animals.

Based on the data from Trx1 transgenic mice and other mice that have either up-regulated or down-regulated various antioxidant enzymes, it is clear that the role of oxidative stress in aging seems to be more complex than the oxidative stress theory describes. The effects of Trx1 overexpression (and oxidative stress) could be different between young and old mice. The protection against oxidative stress and changes in redox-sensitive signaling, for example, inflammatory signaling pathways, by Trx1 could be beneficial in young animals. However, our study has also indicated that reduced oxidative stress and changes in redox-sensitive signaling, for example, apoptosis signaling pathways, by Trx1 could play important roles in cancer growth, which has more deleterious effects in older animals.

Redox change in different cellular compartments is another important factor to be considered when studying the role of oxidative stress in aging. Since mitochondria generate most of the endogenous ROS, overexpressing Trx in mitochondria (Trx2) could show more significant effects on oxidative stress and longevity. Although Trx1 overexpression reduced the oxidative damage to proteins and lipids, DNA oxidation was not reduced by Trx1, which could be another reason why Trx transgenic mice had an increased incidence of cancer. This possibility could be supported by a recent study by Schriner *et al.*[52] which reported that the overexpression of catalase in mitochondria significantly extended lifespan and reduced the incidence of some cancers in mice, which is the only mouse model to support the oxidative stress theory of aging. In addition, Widder* et al.*[85] found beneficial roles of overexpressing Trx in the mitochondria (Tg^hTrx2^ mice) on the development of vascular dysfunction and hypertension, which are common age-related pathological changes in humans. Another study also showed the beneficial effects of Trx2 overexpression on endothelial functions and protection against atherosclerosis development [86]. Therefore, it is of interest to examine if overexpression of Trx2 could attenuate aging and/or age-related pathology in mice. Another important cellular compartment is the nucleus where an elegant study done by Go *et al.*[87] demonstrated that transgenic mice overexpressing Trx1 in the nucleus (NLS-hTrx1Tg) had an increased mortality by influenza H1N1, which was associated with increased inflammatory signaling. These results are contrary to the work with Trx1 (cytosolic) and Trx2 transgenic mice that showed protection against various stresses [86,88]. This is additional evidence supporting the notion that compartmentalized redox regulation is a key component of redox signaling and cellular functions [87]. Therefore, the changes in redox state in different compartments of cells, that is, the mitochondria, cytosol and nucleus, could control the activity of redox-sensitive signaling in cells differently.

These results can potentially provide two interesting scenarios regarding Trx, cancer and aging: 1) overexpressing Trx in the mitochondria could play a more important role on lifespan than in the cytosol, which is similar to the results of the mCAT mice studies (overexpressing catalase in the mitochondria increased lifespan, but overexpressing catalase in the nucleus or the cytosol had no effect); and 2) down-regulating Trx in the cytosol could play important roles in suppressing cancer development, which may have beneficial effects in older animals. These paradoxical, but intriguing, scenarios could indicate that changes in the redox state in the cytosol and mitochondria attenuate lifespan through different mechanisms, for example, protection of the mitochondria against oxidative stress and reduced age-related pathology, such as cancer.

Based on these ideas, we are currently examining the effects of overexpressing Trx2 and/or down-regulating Trx1 on oxidative stress, redox status, redox-sensitive signaling, age-related diseases (especially cancer), and aging using Trx2 transgenic (Tg) and Trx1 knockout (KO) mice. Our ongoing study showed Trx2Tg mice that overexpress Trx2 in all tissues during aging showed less ROS production in mitochondria, less oxidative stress, and had a slight extension of lifespan in the earlier part of life. When we test the effects of reduced levels of thioredoxin in cytosol or mitochondria on aging, we may observe the reverse effects, that is, life-extension and reduction of cancer in the Trx1KO mice, while the Trx2KO mice showed little effects on lifespan but showed impaired mitochondrial function [89,90]. We are also testing if overexpressing Trx2 along with down-regulating Trx1 could show additive anti-aging effects by using mice that up-regulate Trx in the mitochondria and down-regulate Trx in the cytosol (Trx2Tg x Trx1KO mice). The results from this study will allow us to determine if overexpression of Trx in the mitochondria and down-regulation of Trx in the cytosol throughout life play important roles in aging and age-related pathology through common or independent pathways.

Based on our current preliminary data, we predict that overexpressing Trx2 will provide protection against oxidative stress in the mitochondria and delay aging, down-regulating Trx1 will provide protection against tumorigenesis by enhanced apoptosis or reduced cell proliferation to delay aging, and overexpression of Trx2 combined with down-regulation of Trx1 will show a greater anti-aging effect and reduction of pathology than overexpression of Trx2 or down-regulation of Trx1 would alone.

## Conclusions

Although the Free Radical or Oxidative Stress Theory of Aging has been one of the most popular theories in aging research over the past several decades, the results generated from our lab and others have raised more questions about the oxidative stress theory than provided answers. These results seriously call into question the role of oxidative damage/stress in the aging process in mammals, requiring us to make significant modifications to the theory of oxidative stress in aging in order to understand the relationship between aging and the regulation of oxidative stress. The results generated from our lab (Trx1Tg, Trx1KO, Trx2Tg) and others (for example, mCAT mice, NLS-hTrx1Tg mice and Tg^hTrx2^ mice) could suggest that: 1) changes in oxidative stress and redox state in the cytosol, mitochondria or nucleus might play different roles in the aging process and in age-related diseases; 2) the role of oxidative stress and redox state could have different pathophysiological consequences in different tissues/cells, for example, mitotic vs. post-mitotic; 3) oxidative stress could have different pathophysiological impacts in young and old animals; and 4) the pathophysiological roles of oxidative stress and redox state could be controlled through changes in redox-sensitive signaling, which could have more diverse effects on pathophysiology than the accumulation of oxidative damage to various molecules. To critically test the role of oxidative stress on aging and age-related diseases, further study is required using animal models that regulate oxidative stress levels differently in each cellular compartment, each tissue/organ, and/or at different stages of life (young, middle, and old) to change redox sensitive signaling pathways.

## Abbreviations

AP-1, Activator protein 1; ASK1, Apoptosis signal-regulating kinase 1; BPRC, BACPAC Resources Center; CHORI, Children’s Hospital Oakland Research Institute; CR, Calorie restriction; GPX, Glutathione peroxidase; GSH, Glutathione; KO, Knockout; MetO, methionine sulfoxid; MsrA, Methionine sulfoxidereductase A; NFκB, Nuclear factor κB; PAPS, 3′-phosphoadenosine-5′-phosphosulfate; Prx, Peroxiredoxin; ROS, Reactive oxygen species; SOD, Superoxide dismutase; Tg, Transgenic; Trx, Thioredoxin; JNK: c-Jun N-terminal Kinase; IACUC: Institutional Animal Care and Use Committee.

## Competing interests

The authors declare that they have no competing interests.

## Authors’ contribution

All the authors contributed equally to this article and have read and approved the final manuscript.
